# Ipsilesional 5 Hz Repetitive Transcranial Magnetic Stimulation for Motor Dysfunction in Subacute Intracerebral Hemorrhage: An Exploratory Trial

**DOI:** 10.1016/j.arrct.2024.100386

**Published:** 2024-11-14

**Authors:** Juan Xiao, Yan Sun, Ze-Jian Liu, Liang Wu, Weijiao Fan, An-Ming Hu

**Affiliations:** a Rehabilitation Center, Beijing Xiaotangshan Hospital, Beijing, China; b Department of Rehabilitation Medicine, Beijing Tiantan Hospital, Capital Medical University, Beijing, China

**Keywords:** Affected cortical region, hemiplegia, Intracranial hemorrhage, rehabilitation, Repetitive transcranial magnetic stimulation (rTMS)

## Abstract

**Objective:**

To explore whether ipsilesional 5 Hz repetitive transcranial magnetic stimulation (rTMS) therapy can improve motor function in patients with intracerebral hemorrhage (ICH) and observe any adverse reactions.

**Design:**

A 4-week randomized, controlled, single-blind (evaluator) trial with a 1-month follow-up.

**Setting:**

A tertiary hospital rehabilitation center.

**Participants:**

Forty-nine patients with first ICH were recruited and randomly separated into an experimental (n=25; 19 men, 6 women) or control group (n=24; 17 men, 7 women), with age range of 30 to 75 years, and mean duration after hemorrhage onset of 47 (range 17-86) days.

**Interventions:**

The experimental group received ipsilesional 5 Hz rTMS therapy and conventional rehabilitation training. The hot spot of the abductor pollicis brevis and tibialis anterior muscles on the affected hemisphere of the brain received 1500 pulses of stimulation each day, for a total of 3000 pulses. The stimulations applied to the affected abductor pollicis brevis and tibialis anterior hot spots were separated by >2 hours. The stimulation frequency was 5 Hz, each sequence lasting 2 seconds, with a sequence interval of 5 seconds, for a total duration of 36 minutes every day. The control group only received conventional rehabilitation training.

**Main Outcome Measures:**

The primary endpoint was the change in Brunnstrom stage. Secondary endpoints included Fugl-Meyer Assessment, Barthel Index, and Berg Balance Scale. All assessments were performed at baseline, after intervention (day 29) and 1 month after intervention (day 60).

**Results:**

Improvements over baseline in all scores at day 29 and 60 were significantly greater in the 5 Hz rTMS group than in the control group. No significant side effects were reported during the experiment and 1 month after the experiment.

**Conclusions:**

Applying 5 Hz high-frequency rTMS to the affected hemisphere within 3 months after the onset of ICH appears safe and may significantly improve motor function and activities of daily living.

Intracerebral hemorrhage (ICH) is one of the leading causes of death and disability in China. With the increasing number of survivors, innovative and effective treatment methods are continuously being developed. Repetitive transcranial magnetic stimulation (rTMS), as a noninvasive stimulation method, has been applied in the treatment of motor and functional disorders caused by stroke in recent years, with significant therapeutic effects.^1^ Low-frequency (≤1 Hz) stimulation inhibits cortical excitability,^2^ whereas high-frequency (≥5 Hz) stimulation excites cortical excitability.^3^

Jang et al^4^ reported a patient in a vegetative state after a right-sided basal ganglia ICH who showed improvement in consciousness after high-frequency rTMS treatment of the unaffected hemisphere at 5 weeks postonset. An animal experimental study^5^ suggested that in mice artificially induced to have an ICH, high-frequency rTMS administered on the day of the hemorrhage and continued once a day for 5 days significantly reduced brain edema and improved neurologic function compared with the sham stimulation group. Functional magnetic resonance imaging confirmed that high-frequency rTMS treatment of the left dorsolateral prefrontal cortex at an average of 52 days postonset of stroke (including infarction and hemorrhage) increased brain functional connectivity and improved cognitive function.^6^

According to the “Evidence-based guidelines on the therapeutic use of repetitive transcranial magnetic stimulation” published in *Clinical Neurophysiology* in 2014^7^ and 2020,^8^ rTMS was mainly used in the treatment of ischemic stroke, particularly with low-frequency stimulation of the unaffected hemisphere. However, there are relatively few studies on the use of high-frequency rTMS in the subacute phase after stroke, and currently, there are no reports specifically on the use of high-frequency rTMS on the affected side in the subacute period after ICH.

Currently, the main side effects of rTMS include headache during treatment, pain and discomfort at the stimulation site, but these reactions could be quickly relieved after the treatment ends. The most serious side effect was epilepsy, which occurred with a very low probability (<0.1%) and mostly at a treatment frequency of ≥15 Hz.^9^^,^^10^ Currently, there are no reports of rTMS inducing bleeding. After cerebral infarction, whether it was high-frequency stimulation of the affected hemisphere or low-frequency stimulation of the unaffected hemisphere, no serious adverse effects (including epilepsy) had been found.^7^ Similarly, we speculated that high-frequency stimulation of the affected hemisphere in patients with ICH was safe and feasible after the bleeding has stopped. High-frequency rTMS directly targets and modulates the corresponding cortical region of the lesion, so we planned to further explore the effect of high-frequency 5 Hz rTMS on motor dysfunction in the subacute period after ICH.

## Materials and methods

### Participants

From 2020 to 2023, patients were recruited from the rehabilitation center. The inclusion criteria were as follows: (1) first occurrence of ICH, with bleeding already stopped and 15 days to 3 months after the onset of disease; (2) age between 30 and 75 years; (3) clear consciousness and able to cooperate with physical examination, scoring, and treatment; and (4) patients and their families willing to receive rTMS treatment and sign the informed consent form. The exclusion criteria were as follows: (1) those with intracranial metal implants, heart pacemakers, or cochlear implants; (2) those with retinal detachment, glaucoma, unstable hypertension, severe headache, or severe dysfunction of the heart, lungs, liver, or kidney; (3) those with severe cognitive, communication, emotional, or restlessness disorders unable to cooperate.

### Experimental design

**Fig 1:**
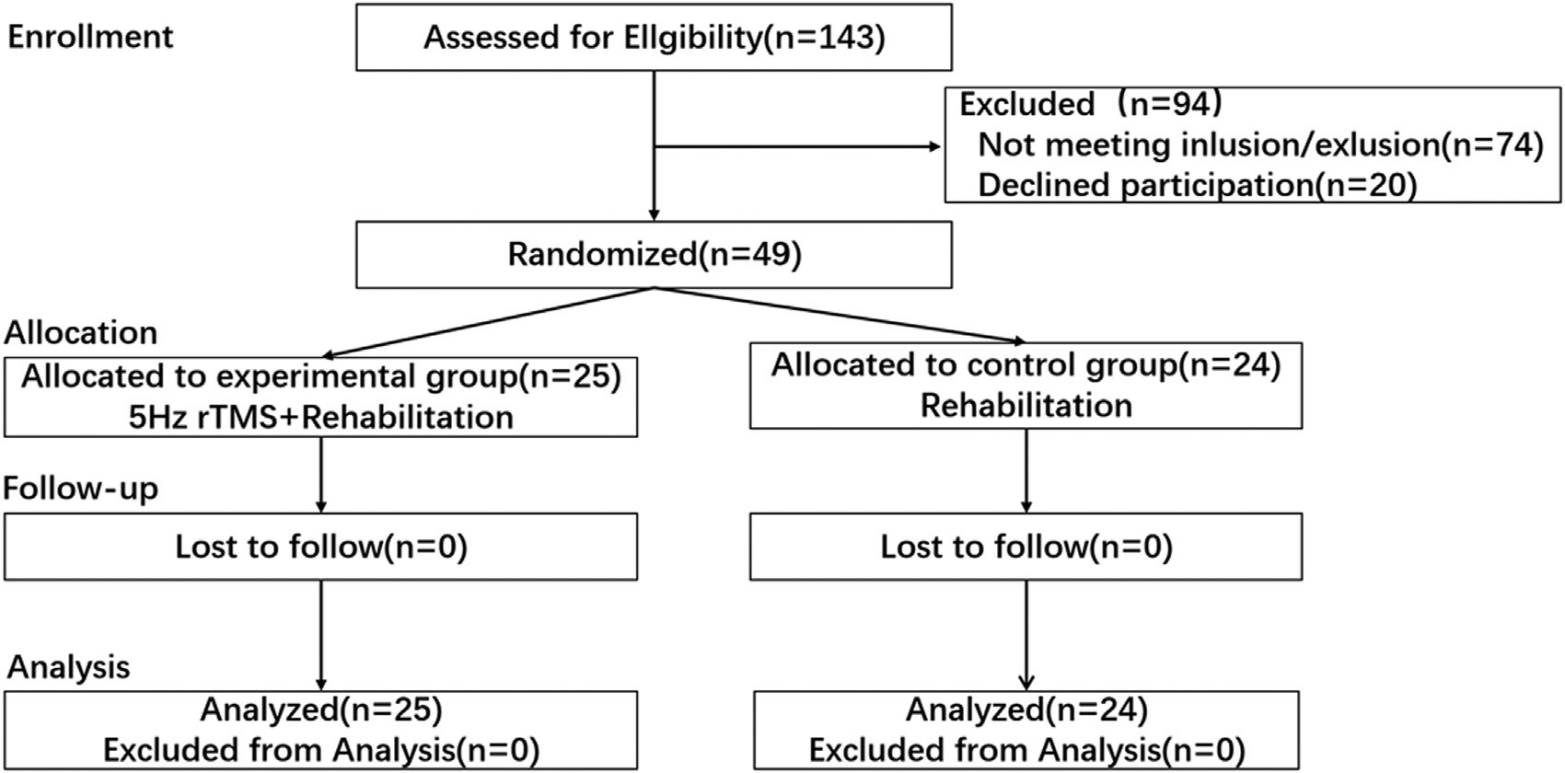
Consort flowchart illustrates recruitment, group allocation, allocation treatment, follow-up and analysis.

This was a single-blinded (the evaluators) randomized controlled trial. This study was performed in accordance with the policies set by the Declaration of Helsinki, and the research procedure was approved by the Ethic Committees of Beijing Xiaotangshan Hospital, Beijing, China (Approval No. 2018-44). Stratified randomization was performed, with gender, lesion location (subcortical/cortical), and side of lesion (left/right) as the stratifying factors, to ensure that the basic information between the 2 groups remained as consistent as possible. A computer random number generator was used to generate the random sequence. The allocation sequence was concealed using sealed, numbered, opaque envelopes. The sample size was calculated to be 42 using the G*Power 3.1.9.7,^11^^,a^ with a detectable change 0.9 (assumed SD, 1.1) of upper limb Brunnstrom stage (BS) or an effect size of 0.82 according to pre-experiment, and a level of significance of 0.05, statistical power of 80% with one-tailed Wilcoxon-Mann-Whitney test. Considering a possible dropout rate of 20% to 30%, a total of 49 participants were included for analysis (fig 1).

During a 4-week period, both groups received conventional rehabilitation treatment, including limb motor function training, cycle training, balance function training, daily living activities training, and neuromuscular electrical stimulation therapy, twice a day for 2 hours each session. These exercises were conducted by therapists who were unaware of the group assignments. In addition, the experimental group received ipsilesional 5 Hz rTMS treatment, with the stimulation site being the motor cortex projection area of the affected limb's abductor pollicis brevis and tibialis anterior muscles.

**Fig 2:**
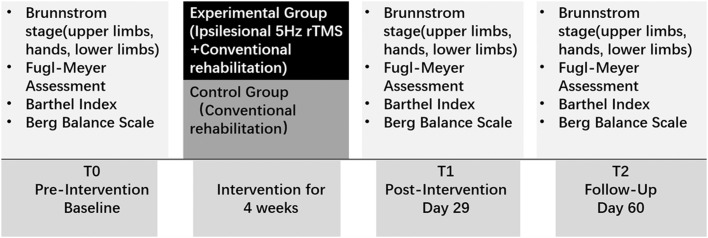
Experimental design.

The assessments were measured before treatment, immediately after treatment (day 29) and 1 month after treatment (day 60) by a rehabilitation therapist who was unaware of the group allocation (fig 2). Primary outcome measure included the BS.^12^ Secondary outcome measures included Fugl-Meyer Assessment (FMA),^13^ Barthel Index (BI),^14^ and Berg Balance Scale (BBS).^15^ All participants were followed up during the treatment period, as well as 1 month after treatment, to observe any adverse reactions such as epilepsy, headache, dizziness, tinnitus, increased blood pressure, and tinnitus.

### Determination of the resting motor threshold

The rTMS device^b^ had a maximum output intensity of 3T, circular dynamic air-cooled coil (diameter 120 mm), stimulation depth of 3 cm, and magnetic field area of 2 cm^2^. During treatment, patients were instructed to lie comfortably and quietly on their backs, avoiding head movement as much as possible during the treatment process. The midpoint of the circular coil was placed in contact with the skull surface near the M1 region of the stimulated hemisphere. The position of the coil was adjusted by changing the tilt angle, first using the maximum intensity output to find the “hot spot” where the maximum motor evoked potential (MEP) amplitude could be elicited from the affected tibialis anterior and abductor pollicis brevis muscles. After identifying the hot spot, resting motor threshold was obtained by gradually reducing the TMS intensity from maximum until a minimal intensity that still evoked an MEP amplitude of at least 50 uV was reached. Using a disk-shaped electrode to record MEP from the abductor pollicis brevis and tibialis anterior muscles, the reaction was recorded via an electromyogram.^c^ Resting motor threshold was defined as follows: in a muscle relaxation state, with the smallest TMS stimulation intensity, stimulating 5 times continuously, and at least 3 times evoking an MEP amplitude of no <50 uV.^16^ If there was no response from the affected limb using maximum stimulation intensity for 3 consecutive stimulations, MEP was defined as absent.^17^

### rTMS application

The operator placed the circular coil in contact with the skull, applying it to the corresponding hot spot of the abductor pollicis brevis and tibialis anterior muscles on the affected hemisphere of the brain. If the MEP was absent in the affected limb's abductor pollicis brevis or tibialis anterior muscle, the symmetrical position that could elicit the MEP in the contralateral muscle was used as the treatment target. The treatment parameters were as follows: an output intensity of 80% of the resting motor threshold (if the MEP was absent in the affected limb's abductor pollicis brevis or tibialis anterior muscle, the intensity was set at 60% of the maximum output), the hot spot of the abductor pollicis brevis and tibialis anterior muscles on the affected hemisphere of the brain received 1500 pulses of stimulation each day, for a total of 3000 pulses. The stimulations applied to the affected abductor pollicis brevis and tibialis anterior hot spots were separated by >2 hours. The stimulation frequency was 5 Hz, with 10 pulses per sequence, for a total of 300 sequences, each sequence lasting 2 seconds, with a sequence interval of 5 seconds, for a total duration of 36 minutes. The treatment was administered for 4 consecutive weeks, 5 days a week.

The rTMS protocol used in this study was based on the safety guidelines for rTMS applications.^18^

### Statistical analysis

Using IBM SPSS statistics version 26^d^ software package for data analysis. The unordered categorical variables (gender, lesion location, left/right) were compared using Fisher exact test. The within-group comparisons (include BS, FMA, BI, and BBS) before treatment, after treatment and 1 month after treatment were conducted using Kruskal-Wallis one-way analysis of variance test with post hoc multiple comparisons, whereas the between-group comparisons before and after treatment were conducted using Mann-Whitney *U* test to evaluate the effectiveness of rTMS. A *P* value ≤ .05 was considered statistically significant.^19^ Cohen *d* effect sizes were reported based on the Mann-Whitney *U* test statistic. A website of “https://www.psychometrica.de/” was used for effect size calculations. The suggested interpretations for the magnitude of the effect size are as follows: small effect (about 0.2), medium effect (about 0.5), and large effect (≥0.8).^20^

## Results

A total of 49 participants completed the treatment, and no significant discomfort was reported during the treatment period. Demographic data are outlined in table 1. There were no statistically significant differences between the 2 groups in age, gender, distribution of injury site (cortical and subcortical), affected hemisphere (left and right), time of onset and absence of MEP of hemiplegic abductor pollicis brevis and tibialis anterior muscle. Before treatment, there were no statistically significant differences between the 2 groups in all BS, FMA, BI, and BBS (table 2).

**Table 1:** Demographic and clinical data

Characteristics	Experimental Group (n=25)	Control Group (n=24)	*P*
Sex (m/f)	19/6	17/7	.754
Age, y	53.9 (14.9)	54.0 (13.0)	.976
Lesion location (subcortical/cortical)	21/4	21/3	1.000
Side of lesion, l/r	14/11	13/11	1.000
Days after hemorrhage onset	51.4 (19.4)	42.3 (24.3)	.159
Absence/existence of MEP of hemiplegic APB	23/2	22/2	1.000
Absence/existence of MEP of hemiplegic TA	19/6	20/4	.725

The data are presented as mean (standard deviation) or number/total number.

Abbreviations: APB, abductor pollicis brevis; f, female; l, left; m, male; r, right; TA, tibialis anterior.

**Table 2:** Primary and secondary outcome measures in patients between groups and over time

					Change From Baseline				Change From Day 29			
Measures	rTMS Group (n=25)	Control Group (n=24)	*P* Value*	*d* Effect Size	rTMS Group (n=25)	Control Group (n=24)	*P* Value*	*d* Effect Size	rTMS Group (n=25)	Control Group (n=24)	*P* Value*	*d* Effect Size
	Median (IQR)	Median (IQR)			Median (IQR)	Median (IQR)			Median (IQR)	Median (IQR)		
Primary outcomes												
BS of arm												
Baseline	1 (1)	1 (0)	.212	0.271								
Day 29	2 (2)	1 (1)	.006	0.797	1 (1.5)	0 (1)	.012	0.696				
Day 60	3 (2)	2 (2)	.000	1.111	2 (1)	1 (1)	.000	1.077	1 (1)	0 (1)	.045	0.520
BS of hand												
Baseline	1 (1)	1 (0)	.264	0.242								
Day 29	2 (2)	1 (0.75)	.016	0.655	0 (1.5)	0 (0)	.012	0.613				
Day 60	3 (3)	1 (1)	.003	0.884	1 (2)	0 (0)	.001	1.016	0 (1)	0 (0)	.010	0.570
BS of leg												
Baseline	2 (3)	1 (2)	.300	0.280								
Day 29	4 (1)	2.5 (2)	.004	0.862	1 (2)	0 (0)	.005	0.747				
Day 60	4 (1)	3 (1.75)	.000	1.255	1 (1)	0 (1)	.001	0.972	0 (1)	0 (0)	.067	0.420
UE-FMA												
Baseline	4 (10)	4 (0.75)	.315	0.236								
Day 29	14 (21.5)	4 (9.75)	.007	0.797	10 (15)	0 (8)	.004	0.843				
Day 60	27 (20)	8 (12.75)	.000	1.337	22 (13)	4 (7.75)	.000	1.506	8 (8)	3 (2)	.000	1.907
LE-FMA												
Baseline	9 (17)	4 (15.75)	.411	0.221								
Day 29	21 (3.5)	13.5 (15.75)	.002	0.949	3 (15)	0 (0.75)	.002	0.953				
Day 60	30 (4.5)	18 (17)	.000	1.545	13 (14.5)	4 (3.75)	.000	1.592	8 (3)	4 (2.75)	.000	1.679
Secondary outcomes												
BBS												
Baseline	8 (17.5)	5.5 (15)	.762	0.086								
Day 29	21 (28)	19.5 (26.75)	.029	0.652	16 (14)	10 (12.75)	.004	0.910				
Day 60	40 (21)	25 (26.5)	.002	0.968	31 (14.5)	16 (12.75)	.000	1.298	9 (10)	6 (1.75)	.015	0.737
BI												
Baseline	30 (20)	20 (20)	.161	0.405								
Day 29	50 (22.5)	35 (37.5)	.003	0.953	25 (22.5)	5 (13.75)	.001	1.032				
Day 60	65 (27.5)	45 (30)	.004	0.914	35 (22.5)	20 (10)	.001	1.053	15 (15)	10 (7.5)	.500	0.189

Data are expressed as the median (IQR). Between-group comparisons were calculated using Mann-Whitney *U* test.

Abbreviation: LE-FMA, lower extremity-Fugl-Meyer Assessment.

⁎ Mann-Whitney *U* test.

Seventy-four candidates were excluded, and the reasons included: 36 candidates were excluded because of the onset time exceeding 3 months when they were enrolled; 9 patients were excluded because they were >75 years old; 2 patients were <30 years old; 12 patients had severe speech and cognitive impairment; 15 patients were excluded from observation because of their short hospital stay and dropout.

All participants were followed up at 1 month after treatment and no side effects such as epilepsy, headache, dizziness, tinnitus, increased blood pressure, or tinnitus were reported.

### Primary outcomes

Table 2 summarizes all the measurements over time. The change in BS of arm from baseline to day 60 was higher and statistically significant in the rTMS than the control group (median, 2 [interquartile range (IQR), 1] vs 1 [IQR, 1], respectively; *P*=.000, *d*=1.077). The change in BS of hand from baseline to day 60 was higher and statistically significant in the rTMS than the control group (median, 1 [IQR, 2] vs 0 [IQR, 0], respectively; *P*=.001, *d*=1.016). The change in BS of leg from baseline to day 60 was higher and statistically significant in the rTMS than the control group (median, 1 [IQR, 1] vs 0 [IQR, 1], respectively; *P*=.001, *d*=0.972).

### Secondary outcomes

The change in FMA-upper extremity (FMA-UE) from baseline to day 60 was higher and statistically significant in the rTMS than the control group (median, 22 [IQR, 13] vs 4 [IQR, 7.75], respectively; *P*=.000, *d*=1.506). The rTMS group resented a minimal clinically important difference (MCID) in the FMA-UE (change of at least 12.4 points^21^), increasing by 22 points on day 60 (the control group did not obtain this). The change in FMA-lower extremity from baseline to day 60 was higher and statistically significant in the rTMS than the control group (median, 13 [IQR, 14.5] vs 4 [IQR, 3.75], respectively; *P*=.000, *d*=1.592).

The change in BBS from baseline to day 60 was higher and statistically significant in the rTMS than the control group (median, 31 [IQR, 14.5] vs 16 [IQR, 12.75], respectively; *P*=.000, *d*=1.298). Both groups exhibited a MCID in BBS, defined as a change of at least 5 points.^22^ Specifically, in the rTMS group, the BBS score increased by 16 on day 29 and by 31 points on day 60, compared with 10 on day 29 and 16 points on day 60 in the control group.

The change in BI from baseline to day 60 was higher and statistically significant in the rTMS group than the control group (median, 35 [IQR, 22.5] vs 20 [IQR, 10], respectively; *P*=.001, *d*=1.053). In terms of achieving the MCID in BI, both groups surpassed the MCID threshold of 1.85 for stroke patients^23^ from their baseline scores on day 29 and 60. In detail, in the rTMS group, the BI increased by 25 on day 29 and 35 points on day 60, compared with 5 on day 29 and 20 points on day 60 in the control group.

### Subgroup analysis, participants with BS I

#### Primary outcomes

**Fig 3:**
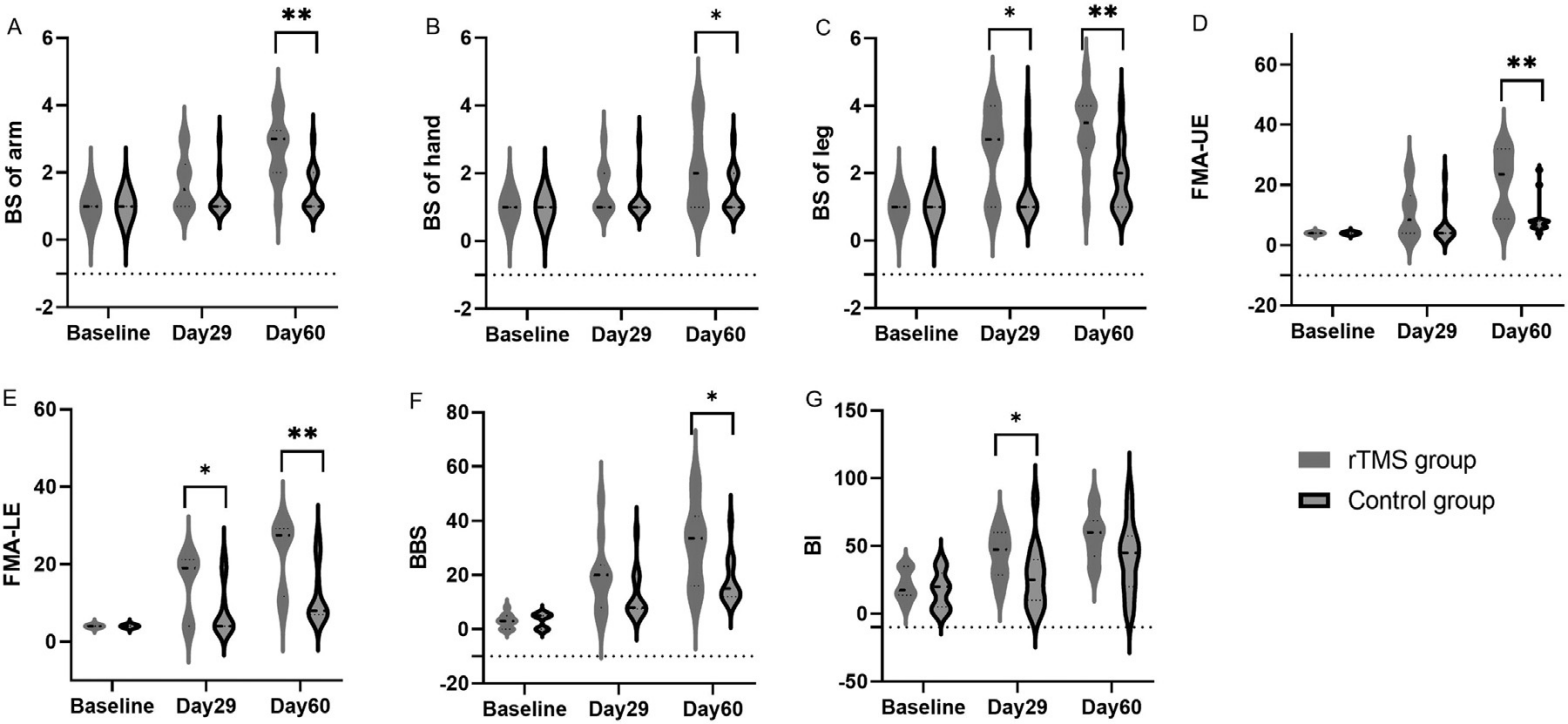
Violin plot of measurements over time in patients with BS I of hemiplegic extremities: BS of arm (A), BS of hand (B), BS of lower limb (C), FMA-UE (D), FMA-LE (E), BBS (F), and BI (G). Abbreviation: FMA-LE, FMA-lower extremity. *P<.05, **P<.001, versus control group (Mann-Whitney *U* test).

Table 3 and figure 3 summarizes all the measurements of participants with BS I over time. In patients with BS I of affected limbs, the change in BS of arm from baseline to day 60 was higher and statistically significant in the rTMS than the control group (median, 2 [IQR, 1.25] vs 0 [IQR, 1], respectively; *P*=.003, *d*=1.431). The change in BS of hand from baseline to day 60 was higher and statistically significant in the rTMS than the control group (median, 1 [IQR, 2.25] vs 0 [IQR, 0], respectively; *P*=.011, *d*=0.961). The change in BS of leg from baseline to day 60 was higher and statistically significant in the rTMS than the control group (median, 2.5 [IQR, 1.25] vs 1 [IQR, 1.5], respectively; *P*=.007, *d*=1.294).

**Table 3:** Primary and secondary outcome measures in patients with Brunnstrom stage 1, comparisons between groups and over time

					Change From Baseline				Change From Day 29			
Measures	rTMS Group (n=10)	Control Group (n=13)	*P* Value*	*d* Effect Size	rTMS Group (n=10)	Control Group (n=13)	*P* Value*	*d* Effect Size	rTMS Group (n=10)	Control Group (n=13)	*P* Value*	*d* Effect Size
	Median (IQR)	Median (IQR)			Median (IQR)	Median (IQR)			Median (IQR)	Median (IQR)		
Primary outcomes												
BS of arm												
Baseline	1 (0)	1 (0)	1.000	0.000								
Day 29	1.5 (1)	1 (0)	.092	0.594	0.5 (1.25)	0 (0)	.092	0.594				
Day 60	3 (1)	1 (1)	.003	1.431	2 (1.25)	0 (1)	.003	1.431	1 (0.5)	0 (0.5)	.006	1.209
BS of hand												
Baseline	1 (0)	1 (0)	1.000	0.000								
Day 29	1 (1)	1 (0)	.093	0.535	0 (1)	0 (0)	.093	0.521				
Day 60	2 (2)	1 (0)	.011	0.961	1 (2.25)	0 (0)	.011	0.961	0 (1)	0 (0)	.014	0.714
BS of leg												
Baseline	1 (0)	1 (0)	1.000	0.000								
Day 29	3 (3)	1 (2)	.042	0.842	2 (3)	0 (1.5)	.042	0.842				
Day 60	3.5 (1)	2 (2)	.007	1.294	2.5 (1.25)	1 (1.5)	.007	1.294	0.5 (1)	0 (0.5)	.158	0.507
UE-FMA												
Baseline	4 (0)	4 (0)	1.000	0.000								
Day 29	8.5 (12.5)	4 (0)	.102	0.579	4.5 (12.5)	0 (0)	.102	0.579				
Day 60	23.5 (23.25)	8 (2)	.002	1.695	19.5 (23.25)	4 (2)	.002	1.695	7.5 (7.75)	3 (2)	.000	2.36
LE-FMA												
Baseline	4 (0)	4 (0)	1.000	0.000								
Day 29	19 (17.25)	4 (9.5)	.029	0.926	15 (17.25)	0 (9.5)	.029	0.926				
Day 60	27.5 (17.5)	8 (11)	.002	1.638	23.5 (17.5)	4 (11)	.002	1.638	7.5 (2.25)	4 (1.5)	.000	2.313
Secondary outcomes												
BBS												
Baseline	3 (5)	5 (5)	.605	0.221								
Day 29	20 (15.75)	8 (11)	.186	0.579	15.5 (15.25)	5 (13.5)	.113	0.699				
Day 60	33.5 (25.75)	15 (12)	.018	1.109	30 (23)	11 (14)	.012	1.23	12.5 (7.25)	5 (1.5)	.001	1.755
BI												
Baseline	17.5 (21.25)	20 (25)	.530	0.261								
Day 29	47.5 (31.25)	25 (30)	.043	0.926	22.5 (13.75)	5 (7.5)	.003	1.557				
Day 60	60 (26.25)	45 (37.5)	.093	0.745	35 (21.25)	20 (20)	.036	0.961	15 (20)	10 (7.5)	.590	0.221

Data are expressed as the median (IQR). Between-group comparisons were calculated using Mann-Whitney *U* test.

Abbreviation: LE-FMA, lower extremity-Fugl-Meyer Assessment.

⁎ Mann-Whitney *U* test.

#### Secondary outcomes

In patients with BS I of affected limbs, the change in FMA-UE from baseline to day 60 was higher and statistically significant in the rTMS than the control group (median, 19.5 [IQR, 23.25] vs 4 [IQR, 2], respectively; *P*=.002, *d*=1.695). The rTMS group resented a MCID in the FMA-UE (change of at least 12.4 points^21^), increasing by 15 points on day 29 and 23.5 on day 60 (the control group did not obtain this). The change in FMA-lower extremity from baseline to day 60 was higher and statistically significant in the rTMS than the control group (median, 23.5 [IQR, 17.5] vs 4 [IQR, 11], respectively; *P*=.002, *d*=1.638).

In patients with BS I of affected limbs, the change in BBS from baseline to day 60 was higher and statistically significant in the rTMS than the control group (median, 30 [IQR, 23] vs 11 [IQR, 14], respectively; *P*=.012, *d*=1.23). Both groups demonstrated a MCID in BBS, which was defined as an increase of at least 5 points.^22^ In detail, the rTMS group experienced a notable enhancement in their BBS scores, with a 15.5-point rise on day 29 and a remarkable 30-point jump on day 60. In contrast, the control group showed a more modest progression, with a 5-point gain on day 29 and an 11-point increase on day 60. The change in BI from baseline to day 60 was higher and statistically significant in the rTMS group than the control group (median, 35 [IQR, 21.25] vs 20 [IQR, 20], respectively; *P*=.036, *d*=0.961).Both groups exhibited a MCID in BI, defined as a change of at least 1.85 points for stroke patients.^23^ Specifically, in the rTMS group, the BI increased by 22.5 on day 29 and 35 points on day 60, compared with 5 on day 29 and 20 points on day 60 in the control group.

## Discussion

This study was our first exploration of the effect of high-frequency rTMS on upper and lower limb motor function recovery in the subacute stage of ICH. Both rTMS combined with conventional rehabilitation treatment and conventional rehabilitation treatment alone could improve balance function and ability of activities of daily living, and both had clinical significance. Both rTMS combined with conventional rehabilitation treatment and conventional rehabilitation treatment alone could increase the FMA-UE, but only rTMS combined with conventional rehabilitation treatment achieved clinical significance. This shows that rTMS combined with conventional rehabilitation treatment can better improve dysfunction than conventional rehabilitation treatment alone.

After central nervous system injury, the nervous system has plasticity, and the most fundamental, important, and active form of plasticity is synaptic plasticity, which is the foundation of human learning and memory, as well as diseases, aging, nervous growth, development, and repair. In the early stages of ICH, neural plasticity is strong, and the first 3 months after the onset of disease is the golden period for functional recovery. From 3 to 6 months after the onset of disease, functional recovery enters a subgolden period. After 6 months, functional recovery enters a plateau period.^24^ Because of the limited volume of the brain, increased intracranial pressure after ICH and local necrosis and rupture of neurons cause the release of inflammatory factors, resulting in edema around the bleeding site and compression of local microvessels, leading to ischemic hypoxia changes in the surrounding cells and the appearance of pathologic changes similar to ischemic penumbra.^25^ Early intervention can rescue ischemic penumbra and avoid neuronal apoptosis, thereby improving function and prognosis.

Brain hemorrhage can lead to an increase in brain parenchyma volume and cerebral edema, resulting in increased intracranial pressure and a high risk of epilepsy.^26^ Previous studies mainly focused on the treatment of stroke with high-frequency on the ipsilesional or low-frequency stimulation on the contralesional motor cortices, without specifically studying the use of high-frequency stimulation on the ipsilesional motor cortices in patients with brain hemorrhage.^7^^,^^8^ In theory, high-frequency rTMS can increase brain excitability, which may increase intracranial pressure and the risk of epilepsy in patients with brain hemorrhage. However, animal experiment has found that treating mice with artificial brain hemorrhage with high-frequency rTMS for 5 consecutive days did not increase intracranial pressure but instead reduced cerebral edema and promoted neural repair.^5^ In addition, a study by Sasaki et al^27^ found that high-frequency rTMS treatment starting 11 days after stroke improved lower limb motor dysfunction in patients with stroke (including both hemorrhage and infarction), including 7 patients with brain hemorrhage who underwent high-frequency rTMS treatment without any adverse effects. A study by Park et al^16^ suggested that bilateral high-frequency (10 Hz) rTMS treatment starting from 4.2 to 6.6 weeks after stroke improved swallowing function in patients with stroke (including both hemorrhage and infarction), including 6 patients with brain hemorrhage who underwent high-frequency rTMS treatment without any adverse effects.

This study treated 25 patients with ICH with 5 Hz rTMS, and no serious adverse reactions such as epileptic seizures occurred. This indicates that rTMS intervention within 3 months after ICH can significantly improve motor function without serious adverse effects. Previous reports have shown that the stimulating frequency that can cause epileptic seizures, when the stimulus does not exceed 120% of the resting motor threshold and no antidepressants are taken, is between 15 and 25 Hz.^10^^,^^28^, ^29^, ^30^ This study used 5 Hz rTMS treatment, which is far below above frequencies, and has been proven to be safe, effective, and feasible for treatment.

### Study limitations

This study was associated with several limitations. First, the time span for patient enrollment was relatively long, and it could be further shortened in the future, for instance, by limiting to within 1 month of onset. Second, there was no strict limitation on the bleeding location. Third, our experiment was limited by the lack of more advanced evaluation methods, such as electrophysiology, functional magnetic resonance imaging, near-infrared. Fourthly, the study was limited by the single- (rather than double-)blinded design. Fifthly, there was no specific temporal relationship between rTMS treatment and rehabilitation therapy. Sixthly, we only observed differences between 2 groups after intervention and 1-month after intervention. Hence, long-term efficacy of 5-Hz rTMS is needed to explore in further studies. Seventhly, as ordinal categorical data, the BS requires the use of nonparametric statistical tests, which does not fully use the information provided by the sample data, resulting in a relatively lower statistical power compared with parametric tests. Last, the intervention group received 36 more minutes of rTMS treatment per day than the control group.

## Conclusions

Although more research is needed, applying 5 Hz high-frequency rTMS to the affected hemisphere within 3 months after the onset of ICH appears safe and may significantly improve motor function and activities of daily living.

## Suppliers

a. G*Power 3. 1.9.7; University Kiel, Germany.
b. Pulsed Magnetic Stimulation Device S-100; Shenzhen Yingchi Technology Co, Ltd.
c. Nicolet Viking Quest; Natus Neurology Incorporated.
d. SPSS version 26; IBM.

## Disclosures

The authors declare that they have no known competing financial interests or personal relationships that may have appeared to influence the work reported in this paper.
